# ﻿A new species of the genus *Prosopistoma* Latreille, 1833 (Ephemeroptera, Prosopistomatidae) from Morocco

**DOI:** 10.3897/zookeys.1117.83539

**Published:** 2022-08-15

**Authors:** Majida El Alami, Mokhtar Benlasri, Michel Sartori, Laurent Vuataz, Mohamed Ghamizi

**Affiliations:** 1 Laboratoire Ecologie, Systématique, Conservation de la Biodiversité (LESCB), Unité de Recherche Labellisée CNRST N°18, Université Abdelmalek Essaâdi, Faculté des Sciences, Département de Biologie, B.P.2121 93002, Tétouan, Morocco Université Abdelmalek Essaâdi Tétouan Morocco; 2 Muséum d’Histoire Naturelle de Marrakech, Université Cadi Ayyad, Faculté des Sciences, Semlalia, B.P. 2390, Marrakech, Morocco Université Cadi Ayyad Marrakech Morocco; 3 Museum of Zoology, Palais de Rumine, Place Riponne 6, CH-1005, Lausanne, Switzerland Museum of Zoology, Palais de Rumine Lausanne Switzerland; 4 Department of Ecology and Evolution, Lausanne University, CH-1015, Lausanne, Switzerland Lausanne University Lausanne Switzerland

**Keywords:** High Atlas Mountains, mayfly, North Africa, Western Palearctic

## Abstract

We describe a new species of *Prosopistoma* collected in the High Atlas Mountains of Morocco. *Prosopistomamaroccanum***sp. nov.** appears to be morphologically more similar to the European highly endangered *P.pennigerum* (Müller, 1785) than to the other Maghrebian species, *P.alaini* Bojkova & Soldán, 2015. A gene tree including the few available barcode sequences of Palearctic *Prosopistoma* specimens is provided. Possible affinities with West African species are also discussed.

## ﻿Introduction

Prosopistomatidae is an Old-World family of Ephemeroptera with all 29 species belonging to the genus *Prosopistoma* Latreille, 1833. *Prosopistoma* is represented by 15 species in the Oriental region, six in the Afrotropical region, six species in the Palearctic region, and two in the Australasian region (Barber-James et al. 2008, 2013; Barber-James 2009, 2010a; Shi and Tong 2013; Bojkova and Soldán 2015; Balachandran et al. 2016; Schletterer et al. 2016, 2021; Roopa et al. 2017; Kazanci and Türkmen 2018; Boonsoong and Sartori 2019). The six Palearctic species occur in its western region: *Prosopistomapennigerum* (Müller, 1785) (Europe), *Prosopistomaoronti* Alouf, 1977 (Levant), *Prosopistomaorhanelicum* Dalkiran, 2009 (Turkey), *Prosopistomaturcica* Kazanci & Turkmen, 2018 (Turkey), *Prosopistomahelenae* Bojkova & Soldán, 2015 (Middle East), and *Prosopistomaalaini* Bojkova & Soldán, 2015 (Algeria). Thus far, only this last species has been identified from the Maghreb.

*Prosopistomapennigerum* is the most widespread species, with a range from France and Portugal to Russia (Schletterer et al. 2021); currently only three isolated populations in Spain, Albania, and Russia are documented.

The first record of a North African *Prosopistoma* population was made by Gagneur and Thomas (1988) who listed specimens from the Tafna watershed in western Algeria under the name Prosopistoma?pennigerum. This population was described later by Bojkova and Soldán (2015) as the new species *Prosopistomaalaini*. This species is probably extinct in the wild, as documented by Benhadji et al. (2019). Touabay et al. (2002) made the second report of *Prosopistoma* nymphs in Maghrebian rivers, as *Prosopistoma* sp.; they collected their material at Tizguit Wadi in the Moroccan Middle Atlas (El Alami et al. 2022).

In the present study, we describe a new species of *Prosopistoma* based on nymphs from the High Atlas Mountains of Morocco. In addition, distinctive characters of Western Palearctic *Prosopistoma* species are provided.

## ﻿Materials and methods

**Figure 1.**
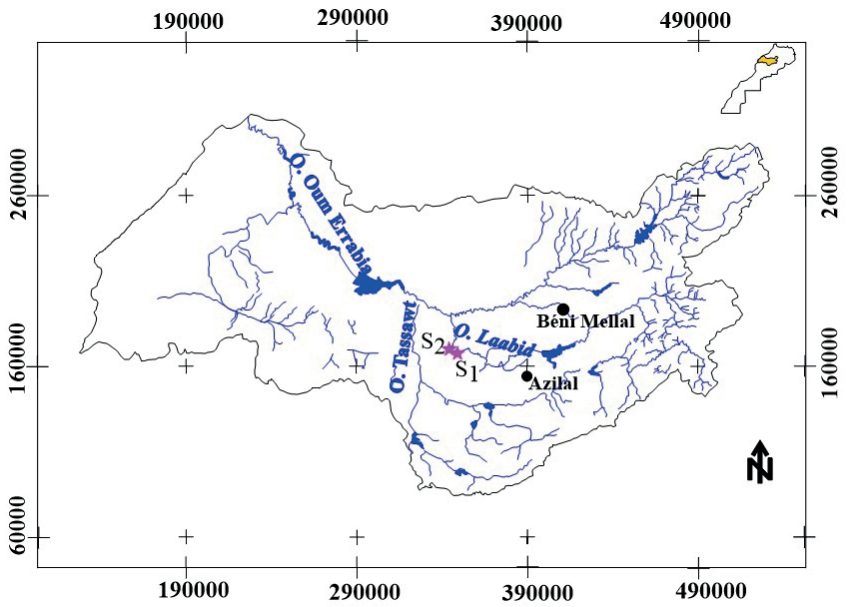
The sampling site localization of *Prosopistomamaroccanum* sp. nov. in Laabid River, Morocco.

The nymphs of *Prosopistomamaroccanum* sp. nov. were collected at two sites on the Oued Laabid, a tributary of the Oum Errbia River which originates in the High Atlas Mountains (Fig. 1). The sampling was performed by Ghamizi team (director of the Museum of Natural Sciences of Marrakech) in 2016 and by Benlasri in 2021. They were subsequently preserved in 95% ethanol for description and DNA extraction. Nymphal dissection was performed in Cellosolve or in 10% KOH, and specimens were mounted on slides with Euparal medium, or the dissected parts of the nymphs were mounted directly in Hoyer’s liquid (Alba-Tercedor 1988).

Several specimens belonging to our new species were processed for DNA extraction, targeting a fragment of the mitochondrial cytochrome c oxidase subunit I gene (COI). Despite numerous attempts, the COI of only one nymph was successfully sequenced. Total genomic DNA was extracted using the BioSprint 96 extraction robot (Qiagen Inc., Hilden, Germany), following the supplier’s instructions. The non-destructive protocol described by Vuataz et al. (2011), which enables post-extraction morphological study of specimens, was implemented. We then amplified a 658-bp fragment at the 5´ end of the COI, corresponding to the standard animal barcode region, using the HCO2198 and LCO1490 primers (Folmer et al. 1994). Polymerase chain reaction (PCR) was conducted in a volume of 25 μl, consisting of 5 μl (unknown concentration) of template DNA, 1.3 μl (10 μM) of each primer, 0.2 μl (25 mM) of dNTP solution (Promega), 5 μl of 5× buffer (Promega) containing 7.5 mM of MgCl_2_, 2.5 μl (25 mM) of MgCl_2_, 1 U of Taq polymerase (Promega), and 9.7 μl of sterile ddH_2_O. Optimized PCR conditions included initial denaturation at 95 °C for 5 min, 40 cycles of denaturation at 95 °C for 30 s, annealing at 50 °C for 30 s, and extension at 72 °C for 40 s, with final extension at 72 °C for 7 min. Purification and automated sequencing was carried out in Microsynth (Balgach, Switzerland). We also included all Palearctic *Prosopistoma*COI sequences available in GenBank and BOLD, resulting in four additional sequences from two species (Table 1). We explored the evolutionary divergence between our new species and the others using the COI genetic distances. The number of parsimony-informative sites and the mean distances between species were calculated in MegaX (Kumar et al. 2018; Stecher et al. 2020) under the Kimura 2-parameter (K80) substitution model (Kimura 1980). We then applied the recently developed species delimitation method ASAP (Assemble Species by Automatic Partitioning; Puillandre et al. 2021) to our COI dataset using the graphical web-interface at https://bioinfo.mnhn.fr/abi/public/asap/asapweb.html. This distance-based method is similar to the popular ABGD (Automatic Barcode Gap Discovery; Puillandre et al. 2012) approach but has the advantage of providing a score that specifies the most likely species delimitation. Pairwise genetic distances were computed under the K80 model, and all other settings were set to default. Finally, we conducted a Bayesian inference gene tree reconstruction in MrBayes v. 3.2.7a (Ronquist et al. 2012), using the best evolutionary model (HKY + Γ) selected in JModelTest 2.1.10 (Darriba et al. 2012) following the second-order Akaike information criterion (AICc; Hurvich and Tsai 1989). We used 11 substitution scheme and six gamma categories, with all other parameters set to default. To accommodate different substitution rates among COI codon positions, we analyzed our data set in two partitions, one with first and second codon positions, and one with third positions (1 + 2, 3). Two independent analyses of four MCMC chains run for one million generations with trees sampled every 1000 generations were implemented, and 200,000 generations were discarded as a burn-in after visually verifying run stationarity and convergence in Tracer v. 1.7.2 (Rambaut et al. 2018). The consensus tree was visualized and edited in iTOL v. 6 (Letunic and Bork 2021). Two *Baetisca* sequences were chosen as the outgroup.

**Table 1.** Codes, origin, and nomenclature of sequences used in molecular study.

Species	Specimen catalogue number	Stage	Locality	GPS coordinates	Date	GenBank ID	GenSeq Nomenclature
*Prosopistomamaroccanum* **sp. nov.**	GBIFCH 00970951	Nymph	Morocco, Oued Laabid, Imdahen	32°8.252 N, 7°1.764 W	6.iii.2016	ON920528	genseq-2 COI
*Prosopistomapennigerum*		Nymph	Albania, Vjosa	40.316°N, 20.030°E	2018	MZ707155	genseq-4 COI
*Prosopistomapennigerum*		Nymph	Russia, Volga, Rzhev	56.260°N, 34.321°E	2018	MZ707154	genseq-4 COI
*Prosopistomaoronti*		Nymph	Israel, En Tina	33.078°N, 35.644°E	27.iii.2019	MN958840	genseq-4 COI
*Prosopistomaoronti*		Nymph	Israel, En Tina	33.078°N, 35.644°E	27.iii.2019	MN958841	genseq-4 COI

The material is deposited in the collections of the Museum of Natural History in Marrakech (**MHNM**), the Laboratoire Ecologie, Systématique, Conservation de la Biodiversité (**LESCB**) in Tétouan, and the Museum of Zoology in Lausanne (**MZL**).

## ﻿Results

### ﻿Molecular analysis

**Figure 2.**
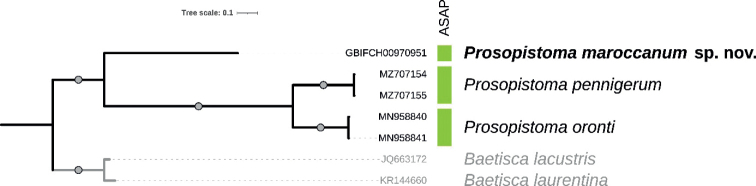
Bayesian majority-rule consensus tree reconstructed from the COI data set of the available Palearctic *Prosopistoma* species. The branch labelled with the GBIF code represents the newly sequenced specimen (species name in bold), other codes correspond to previously published GenBank sequences. Vertical colored boxes indicate species delimitation hypothesis according to the ASAP analysis. The outgroups are represented in grey. Circles on branches indicate Bayesian posterior probabilities of 1.

The COI ingroup data set were 95% complete and included 14% of parsimony informative sites. The missing data resulted from the two *P.pennigerum* sequences from GenBank that lacked 5´ and 3´ ends. All COI gene tree relationships were highly supported, with the *Prosopistomamaroccanum* sp. nov. COI sequence recovered as sister to the other *Prosopistoma* sequences, which were split into two well-supported monophyletic clades according to their species affiliation (Fig. 2). The three *Prosopistoma* species, including *P.maroccanum*, were supported as distinct species in the ASAP analysis. The K80 mean genetic distance between *P.maroccanum* and the other two species ranged from 34.3% (mean distance to *P.pennigerum*) to 35.6% (mean distance to *P.oronti*).

### ﻿Morphological analysis

#### ﻿ Prosopistoma maroccanum

Taxon classificationAnimaliaEphemeropteraProsopistomatidae

El Alami, Benlasri & Sartori sp. nov.

7E5B6A0B-C240-54D1-860B-EFB396008C61

https://zoobank.org/9C03C91D-8E32-47C7-9FD1-DE01A7093072

##### Material examined.

***Holotype***: Morocco • 1 nymph in ethanol; Béni-Mellal Province, Oued Laabid, in Bzou village, 32°6.076'N, 7°2.644'W, 372 m alt., 14 December 2021, coll. M. Benlasri (MZL GBIFCH01119080). ***Paratypes***: Morocco • 1 nymph in ethanol, same data as holotype (MZL GBIFCH01119081); 1 nymph on slide, same data as holotype (MZL GBIFCH00608997; 7 nymphs in ethanol, same data as holotype (LESCB); 1 nymph on slide, same data (LESCB); 3 nymphs in ethanol, same sampling site, 08 May 2021; 4 nymphs in ethanol, same sampling site, 14 October 2021, Coll. M. Benlasri (MHNM) • 4 nymphs in ethanol, Béni-Mellal Province, Oued Laabid, in Imdahen village, 32°8.252'N, 7°1.764'W, 364 m alt., 06 March 2016, coll. H. Hajjani (MZL GBIFCH00980869); 1 nymph on slide, same data (MZL GBIFCH00970951); 5 nymphs in ethanol, same data (LESCB); 5 nymphs in ethanol, same site, 08 May 2021; 7 nymphs in ethanol, same site, 14 October 2021, Coll. M. Benlasri (MHNM).

##### Description.

**Nymph** (in alcohol). Body length 3–4 mm excluding caudal filaments.

**Figure 3.**
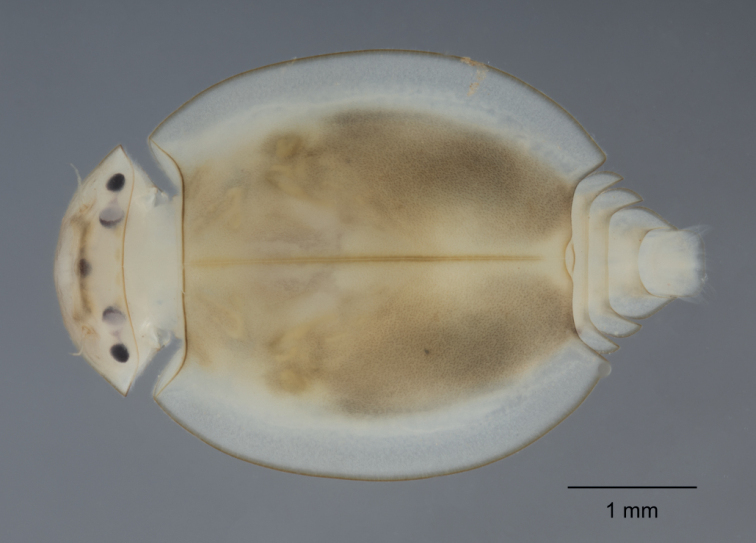
Nymphal dorsal view of *Prosopistomamaroccanum* sp. nov.

Notal shield (carapace) length along median suture 2 mm and total length of body 4.0 mm. Carapace (Fig. 3), wider than long, width/length ratio: 1.15–1.25; head width/length ratio:2.5, head width/carapace width ratio: 0.58–0.60; distance between eyes/head width ratio: ca 0.53 for male nymphs and 0.58 for female nymphs. Carapace flange relatively wide (Fig. 3).

**Figure 4.**
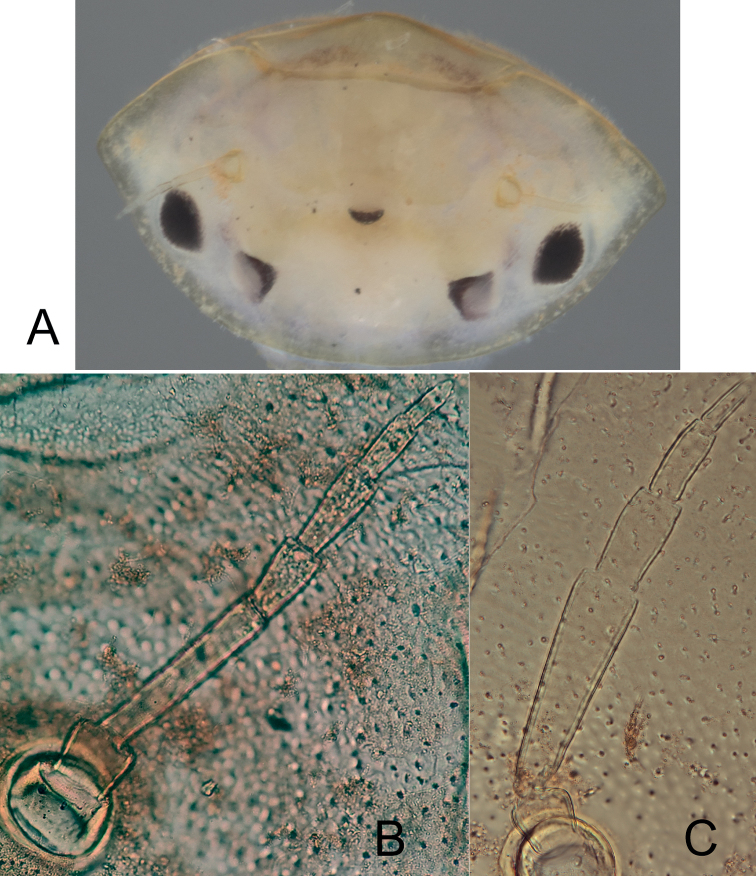
*Prosopistomamaroccanum* sp. nov. **A** head of larva **B** antennae with 7 segments **C** antennae with 6 segments.

***Head*.** Yellowish-brown, with a brownish spot above the median ocellus and between antennae (Fig. 3). Compound eyes roughly oval, blackish, slightly larger than oval lateral ocelli in male nymphs (Fig. 4A). Epicranial suture hardly discernible crossing the middle part of lateral ocelli, and between compound eyes and antennal bases (Fig. 4A) and progressing to the head’s lateral margin. Antennae 7-segmented (including scape and pedicel) in mature nymphs (Fig. 4B), 6-segmented in younger specimens (Fig. 4C); segment III shorter than the total length of segment IV–VI or VII (respectively 0.64× and 0.95×), length of segment III/segments IV–V ratio: 1.18–1.21; antennae not extending beyond the head’s anterior edge.

**Figure 5.**
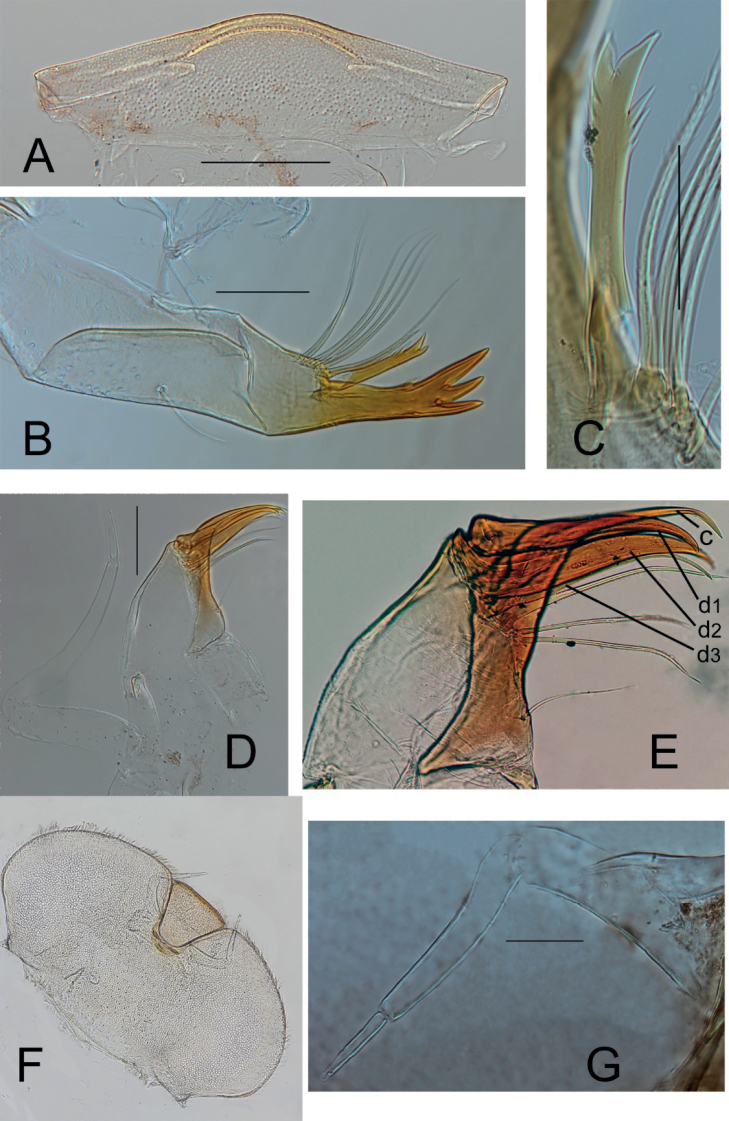
*Prosopistomamaroccanum* sp. nov. **A** labrum (dorsal view) **B** right mandible (ventral view) **C** bristles arising from base of inner canine **D** left maxilla (ventral view) **E** canine (c) and moveable strong dentisetae (d1, d2, d3) **F** labium (ventral view) **G** labial-palp.

Labrum (Fig. 5A) narrow, 3.25× broader than long, anterior margin convex in medial section and straight laterally, anterior margin fringed with fine setae.

Left and right mandibles similar (Fig. 5B). Outer canine distinctly longer and wider than inner canine with three apical teeth, inner one slightly longer than the two others. Outer margin of outer canine with 4–6 micro teeth, inner margin of outer canine with 4 or 5 micro teeth; first distal micro tooth large, conspicuous, more than twice as long and wider than others. Mandible inner canine, one-third shorter than outer canine, rod-shaped, apically bifurcate and forming a pair of elongate and thin pointed teeth with three subapical sharply pointed teeth. 6 or 7 long serrated bristles arising from base of inner canine (Fig. 5C); two first anterior bristles shorter than remaining, and one long simple bristle in the middle of outer margin of mandible.

Maxillae (Fig. 5D, E) crowned by a rigid canine and three moveable, strong dentisetae of subequal length (Fig. 5E). A row of 2 or 3 strong serrated bristles appear below the dentisetae and a single finer bristle on proximal part of sclerotized galea (Fig. 5E). Maxillary palp 3-segmented; segment II long and clearly longer than segment I (1.3–1.5 times) (Fig. 5D).

Labial palps 3-segmented, reaching front margin of labium (Fig. 5F). Labial palp segment II 0.9× length of segment I, segment III 0.5× length of segment II (Fig. 5G).

**Figure 6.**
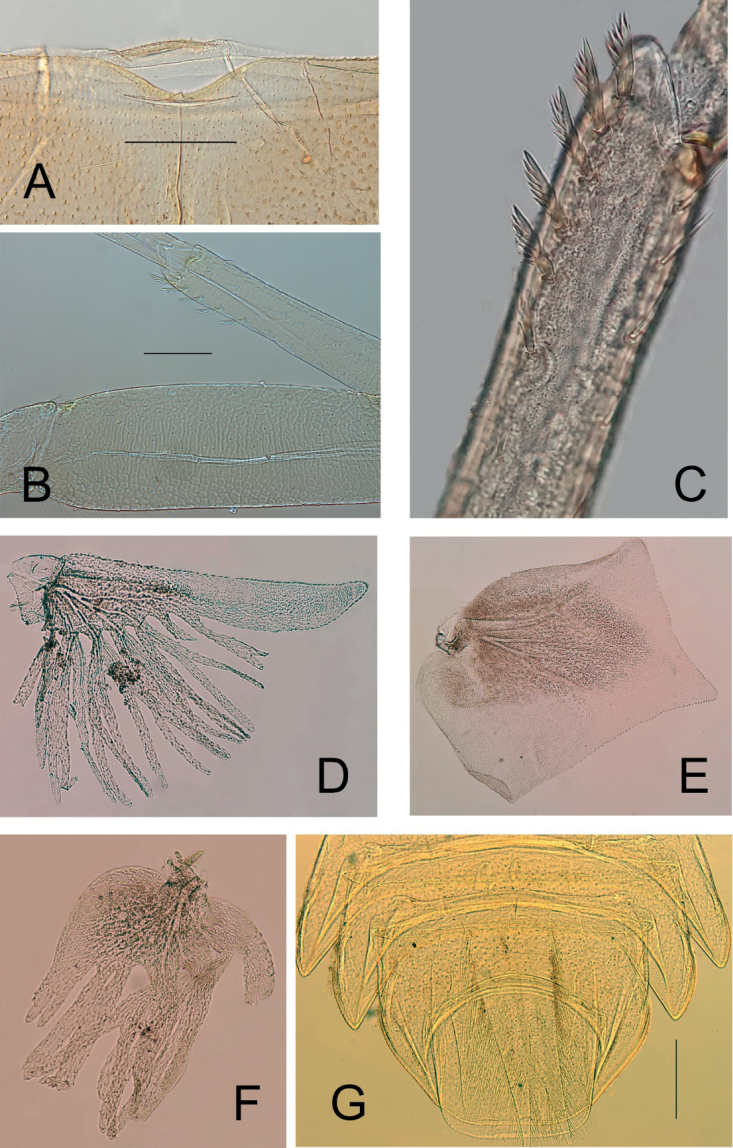
*Prosopistomamaroccanum* sp. nov. **A** posteromedial part of carapace **B** scale-like pattern on femur (ventral view) **C** ventral margin of fore tibia **D** gill I **E** gill II **F** gill III **G** abdominal segments VII–IX.

***Thorax*.** Carapace yellowish brown, with distinct ornamentation (Fig. 6A) and four irregular brownish markings, two on each side of midline of central region of carapace at approximately 0.20× length of carapace from posterior margin of head (Fig. 3); also, there are markings from the anterior end of the carapace at approximately 60° angle to the midline; carapace flange and distal part of carapace transparent but not apparently translucent; relatively wide. Posteromedial part of carapace distinctly concave (Figs 3, 6A).

***Legs*.** Fore femora with reticulate pattern on dorsal surface consisting of scale-like structures more accentuated at the anterior and posterior border (Fig. 6B); dorsal margin of femora with simple and fine setae; ventral margin of fore tibia with 6–8 pectinate setae (Fig. 6C). All tarsal claws sharp, slender, smooth, and without denticles.

***Abdomen*.** Abdominal gills (Fig. 6D–F). Gill I upper portion lamellate with serrated margin, apically slightly asymmetric and rounded; lower section cleaved in numerous filaments with about 6–8 major branches divided into 17–22 filaments (Fig. 6D). Gill II with rectangular lamella (ratio width/length ca 1.10); posterior margin and outer lateral margin concave with short, pointed spines; inner lateral margin convex without spines (Fig. 6E), covering gills III–V appearing with multiple branching filaments, decreasing in number towards gill V (Fig. 6F); gill III with 6 main stems; gill VI conical in shape, very small and unbranched. Abdominal segments VII–IX apparently angular with straight posterior margins; posterolateral projections nearly symmetric, with straight inner margins and relatively pointed apex (Fig. 6G). Segment X rectangular, relatively longer than wider. Caudal filaments, retractile, short, plume-like.

***Imago*.** Not known.

##### Morphological remark.

As already mentioned by Gillies (1954) and Peters (1967), and well documented by Dalkiran (2009) and Schletterer et al. (2016), the number of segments forming the antenna is subject to some variation. In *P.maroccanum*, mature nymphs possess 7 segments, while younger ones have only 6 segments. Therefore, the ratio length of segment III vs length of the remaining segments is to be use cautiously and may have a taxonomic value only when applying to mature nymphs with 7 segments, since this ratio is around 0.95 (subequal) for 6-segmented antennae vs 0.65 (much shorter) for 7-segmented antennae.

##### Diagnosis.

The nymph of *P.maroccanum* sp. nov. appears to be more closely similar to *P.pennigerum* than to *P.alaini* from Algeria (Table 2).

**Table 2.** Morphological discriminant characters between the seven *Prosopistoma* species from the Western Palearctic.

Characters	*P.maroccanum*	*P.pennigerum*	*P.alaini*	*P.oronti*	*P.orhanelicum*	*P.turcica*	*P.helenae*
Antennal segments in mature nymphs (*N*)	7	6	7	5	7	6	6
Antenna reaching/not reaching anterior margin of head	not reaching	reaching	not reaching	not reaching	not reaching	not reaching	reaching
Setae on the right mandible (*N*)	6–7	7–8	8–9	7–9	7	5	5
Subapical teeth on outer margin of outer canine (*N*)	6–7	6–8	5	6–9	7–8	4–5	4–6
Pectinate setae on inner margin of fore tibia (N)	6–8	10–11	10–14	6–7	9–10	7	6–7
Filaments on gill I (N)	17–22	24–28	21–23	12–14	>40	20–22	15–17
Lateral outer margin of gill II	concave	concave	concave	concave	concave	straight	straight
Shape of distal medial margin of carapace	distinctly concave	shallowly concave	straight	distinctly concave	convex	distinctly concave	distinctly concave
Ornamentation of the carapace	distinct	distinct	indistinct	distinct	indistinct	distinct	indistinct
Ratio carapace width / length	1.15–1.25	0.8–0.9	1.1	1.1–1.2	1.1–1.4	1.13	1.2–1.3
Distribution	Morocco	Europe	Algeria	Levant	Turkey	Turkey	Iraq

Indeed, it differs from the latter in several aspects, mainly the distinct ornamentation of the carapace, the lower number of setae on the right mandible (6 vs 8–9), the more numerous subapical teeth on outer margin of the outer canine (6–7 vs 5), the number of maxillary dentisetae (3 vs 4), the distinctly concave distal medial margin of carapace (almost straight in *P.alaini*), and above all by the fewer pectinate setae on the inner margin of fore tibia (7–8 vs 10–14). This last character also separates *P.maroccanum* from *P.pennigerum* (10–11), as well as the number of antennal segments in mature nymphs (7 vs 6), the antenna not reaching the anterior margin of the head (reaching in *P.pennigerum*), and the ratio width/length of the carapace higher in *P.maroccanum* (1.25 vs 0.9). Furthermore, gill I has the apical tip of the dorsal lamina shorter in *P.maroccanum* than in *P.pennigerum*. In addition, the ventral filamentous part possesses a number of main stems which overlap (7–8 vs 8–10) but with a greater number of filaments in *P.pennigerum* (24–28 vs 17–22). *Prosopistomamaroccanum* differs from *P.oronti* mainly by the number of antennal segments (5 in *P.oronti*) and the length of segment II of the antenna shorter compared to the following segments (longer in *P.oronti*). It differs from *P.turcica* mainly by the number of antennal segments (6) and the setation of the right mandible, from *P.orhanelicum* by the fewer subapical teeth on inner margin of outer canine (4–5 vs 6–7), the shape of the distal medial margin of the carapace (convex in *P.orhanelicum*), and the fewer pectinate setae on the inner margin of fore tibiae (9–10 in *P.orhanelicum*). From *P.helenae*, *P.maroccanum* differs in having abdominal segments VII–IX angular (rounded in *P.helenae*) and in the number of antennal segments and length (antenna 6-segmented and reaching the anterior margin of the head in *P.helenae*).

##### Ecology.

**Figure 7.**
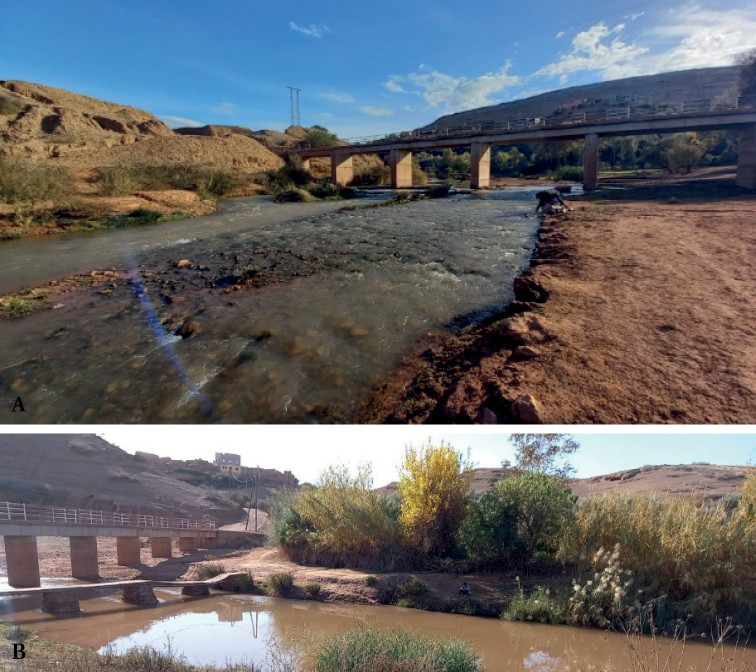
Sampling sites of *Prosopistomamaroccanum* sp. nov. **A** site 1 (Bzou locality) **B** site 2 (Imdahen locality).

*Prosopistomamaroccanum* sp. nov. was collected at two sites in the Laabid River, 117 km from Marrakech. The greatest density, 10 specimens, was recorded in December 2021 at site 2 (Imdahen locality; Fig. 7B). At this site, the bottom structure was composed of 70% pebbles, 20% gravel, and 10% silt. During the sampling campaign, water temperatures were 22.4–23.7 °C, dissolved oxygen 6.57–8.26 mg/l, pH 8.05–8.17, and conductivity 977–999 µS/cm. The channel, about 6 m wide and about 20 cm deep, had a moderate current velocity and turbid water. There was almost no riparian vegetation along the stream banks and no submerged macrophyte cover. *Prosopistoma* species are very sensitive to organic pollution and habitat degradation (Barber-James 2010a). However, there are small villages near the two sampled locations (Fig. 7A, B) which probably increased the turbidity and organic pollution in the water. These impacts probably explain the low density, or the absence of this species, at other sampled sites. The ecological aspects such as microhabitat, nutrition, life history, and phenology of *P.maroccanum* sp. nov. should be further investigated.

## ﻿Discussion

The discovery of a new population and species of *Prosopistoma* in the High Atlas of Morocco is surprising and shows that the biodiversity of Maghrebian mayfly fauna is far from well known. *Prosopistoma* nymphs are so characteristic that we cannot consider their presence has been overlooked by previous studies. The occurrence of *P.maroccanum* sp. nov. is currently limited to a single stream, which may bring arguments that the species should be considered as Critically Endangered based on IUCN criteria. The true identity of the population studied by Touabay et al. (2002) from the Middle Atlas needs also to be explored.

We also investigated the possibility that *P.maroccanum* may represent a relict population of an Afrotropical species. During the African Humid Period, in the late Pleistocene and early Holocene, between roughly 120,000 and 11,500 years before present (de Menocal et al. 2000; Quade et al. 2018), the Sahara was covered by grass savannah, shrubs, and trees, with lakes and running water abundant. In particular, the Tamanrasset paleoriver in western Africa was an important link between the Senegal and Niger watersheds in the south and the streams and rivers of the Atlas Mountains in the north, acting as a pathway for the spread of animals and humans (Skonieczny et al. 2015).

In her extensive and complete review of the Prosopistomatidae worldwide, Barber-James (2010b) studied all described species and several undescribed ones. Among the material, some specimens from West Africa were analysed (J.-M. Elouard collection housed in MZL) and sampled in Guinea, Ivory Coast, Togo, and Sierra Leone (Barber-James 2010b: 298); these she referred to as “African sp. 7”. She recovered all investigated species as belonging to two clades, one called the “*P.variegatum* clade” and the other the “African clade”. Based on the character matrix of Barber-James (2010b: 342–343), *P.maroccanum* would cluster within the “*P.variegatum* clade”, together with *P.pennigerum* and other Palearctic species analysed; African sp. 7 is nested within the “African clade”. We can therefore conclude that *P.maroccanum* is probably Palearctic in origin, separate from the African species, as shown by several characters, among which is the shorter inner incisor of the mandibles, which is subequal to the outer in all species of the “African clade”.

## Supplementary Material

XML Treatment for Prosopistoma maroccanum
